# Cost-effectiveness of a nurse-led intervention to optimise implementation of guideline-concordant continence care: Study protocol of the COCON study

**DOI:** 10.1186/s12912-017-0204-8

**Published:** 2017-02-22

**Authors:** Aaltje P. D. Jansen, Maaike E. Muntinga, Judith E. Bosmans, Bary Berghmans, Janny Dekker, Jacqueline Hugtenburgh, Giel Nijpels, Paul van Houten, Miranda G. H. Laurant, Huub C. H. van der Vaart

**Affiliations:** 1 Amsterdam Public Health research institute, VU University Medical Center, Amsterdam, The Netherlands; 2 Department of General Practice and Elderly Care Medicine, VU University Medical Center, Amsterdam, The Netherlands; 3 Department of Health Sciences and Amsterdam Public Health research institute, Faculty of Earth and Life Sciences, Vrije Universiteit, Amsterdam, The Netherlands; 4 Maastricht University, Maastricht, The Netherlands; Pelvic care Center Maastricht, Maastricht University Medical Centre, Maastricht, The Netherlands; 5 Department of General Practice, University of Groningen; University Medical Center Groningen, Groningen, The Netherlands; 6 Department of Clinical Pharmacology and Pharmacy, VU University Medical Center, Amsterdam, The Netherlands; 7 Zonnehuisgroep Amstelland, Amstelveen, The Netherlands; 8 Faculty of Health and Social Studies, HAN University of Applied Sciences, Nijmegen, The Netherlands; 9 Radboud University Medical Center, Radboud Institute for Health Science, IQ healthcare, Nijmegen, The Netherlands; 10 Department of Obstetrics and Gynaecology, University Medical Centre Utrecht, Utrecht, The Netherlands

**Keywords:** Urinary incontinence, Absorbent products, Nursing, Supplementation, Implementation, Cost-effectiveness, Randomised controlled trial, Community continence care, Primary care

## Abstract

**Background:**

Guidelines on urinary incontinence recommend that absorbent products are only used as a coping strategy pending definitive treatment, as an adjunct to ongoing therapy, or for long-term management after all treatment options have been explored. However, these criteria are rarely met and a significant share of long-term product users could still benefit from therapeutic interventions recommended in guidelines for urinary incontinence. Better implementation of these guidelines can potentially result in both health benefits for women and long-term cost savings for society. The aim of the COCON study is to evaluate the (cost-)effectiveness of a nurse-led intervention to optimise implementation of guideline-concordant continence care in comparison with usual care for urinary incontinent women aged 55 years and over who use absorbent products.

**Methods:**

This randomised clinical trial compares usual care with a nurse-led intervention to optimise implementation of guideline-concordant continence care. Women (anticipated *N* = 160) are recruited in 12 community pharmacies in three Dutch regions, and are eligible for trial entry when they are 55 years and over, community-dwelling and long-term users of absorbent products (≥4 months) reimbursed by health insurance. Measurements are administered at baseline, 3, 6 and 12 months. Primary outcome is severity of urinary incontinence (ICIQ-UI SF); other outcomes include health related quality of life (EQ-5D-5 L), use of absorbent products (in accordance with the recommended criteria in guidelines) (yes/no), and societal costs. Mixed model analysis will be performed to compare (the course) of outcomes between groups. The economic evaluation will be performed from a societal perspective. The implementation process is investigated using the Tailored Implementation for Chronic Diseases (TICD) framework.

**Discussion:**

Results will add to current knowledge of the (cost-)effectiveness of nurse-led primary healthcare to improve guideline-concordant care for older women with urinary incontinence. In addition, the results will provide more insight into care needs and health service utilization of this group of women, as well as into use of absorbent products in accordance with the recommended criteria in guidelines. Finally, results will increase our understanding of the intervention’s uptake and could provide useful insights for future dissemination and sustenance.

**Trial registration:**

Dutch Trial Register NTR4396, registered 13-January-2014

**Electronic supplementary material:**

The online version of this article (doi:10.1186/s12912-017-0204-8) contains supplementary material, which is available to authorized users.

## Background

Urinary incontinence (UI) has been defined as the complaint of any involuntary leakage of urine [1]. In community-dwelling older women, the prevalence of UI is high and increases with age (from 28% in women between 55 and 59 years old to 40% in women over 80), as does severity [2, 3]. The most prevalent UI types in women aged 55 years and older are stress UI, urgency UI and mixed UI. Stress UI occurs during activities that cause a sudden rise in abdominal pressure, such as coughing or sneezing, while urgency UI manifests as an irresistible desire to void, followed almost immediately by loss of urine. Stress UI and urgency UI are estimated to affect 39 and 15% of older women with UI, respectively. Mixed UI consists of symptoms of both urgency and stress, and may affect up to 43% of older women with UI [3]. UI negatively impacts quality of life and role functioning (e.g., work productivity) [4, 5]. People who suffer from UI are more likely to experience adverse health outcomes, such as functional decline and depression, and are more likely to avoid social activities [6, 7].

Evidence based guidelines recommend that treatment is tailored to UI type [8–10]. Treatment has shown to be effective in terms of positively impacting older women’s UI severity, quality of life, and social participation [11–21]. Depressive feelings due to UI’s impact on daily life may improve as well by treatment [22]. In addition, evidence based guidelines recommend that absorbent products are only used as a coping strategy pending definitive treatment, as an adjunct to ongoing therapy, or for long-term management after all treatment options have been explored [8–10]. However, in daily practice these criteria for long-term use of absorbent products are rarely met in female users of 55 years and older in The Netherlands [23–25]. General practitioners (GPs) may not always offer guideline-concordant care to these women, due to, for instance, the unfounded belief that this type of care is likely to be ineffective in this age group [23–25].

Better implementation of the UI guidelines could result in both health benefits for women and long-term cost savings for society, as absorbent products are a main long-term cost driver in continence care [26]: in 2014, 487.800 Dutch users of absorbent products received reimbursement of these products, with each user spending on average € 305 a year, resulting in total costs of almost € 149 million. Better implementation of the guidelines may be facilitated by nurse supplementation in community continence care. Nurse supplementation is defined as adding a nurse to a (primary) care team, who provide services that supplement the care provided by physicians and other relevant health professionals [27]. The aim is to improve quality of care [27]. Evidence suggests that nurse supplementation can produce better health outcomes for patients under the conditions that treatments delivered by nurses are effective and nurses contribute to the improved delivery of those treatments [27]. Nurse supplementation may also positively impact direct healthcare costs, depending on the context in which the care is provided [27].

However, little is known about the effectiveness of nurse supplementation in community continence care. Previous studies that compared nurse supplementation with usual care or no treatment found small to moderate short-term effects, but no long-term effects [28–31]. The evidence on cost-effectiveness is even more limited [28, 30]. A 12-month societal cost-effectiveness analysis suggested that involvement of a nurse specialist was more effective and more expensive than usual care provided by GPs [28]. Furthermore, a decision analytic model suggested that nurse involvement in general practice is likely to reduce incontinence, improve quality of life, and reduce costs [32]. Also, it is unclear in which setting nurse supplementation to community continence care is most appropriate. Past efforts to improve continence care were primarily directed at general practice [28, 29, 32–35], but did not succeed in improving continence care. In The Netherlands, community pharmacies seem an appropriate site to promote guideline-concordant continence care among women who use absorbent products, as absorbent products are distributed to users through pharmacy services and on-site interaction with patients is frequent. In addition, community pharmacies may enable nurses to have clear lines of communication with general practices.

Therefore, this study aims to promote guideline-concordant continence care for women aged 55 years and over using absorbent products by means of nurse supplementation from community pharmacies. The aim of the guideline-COncordant CONtinence care (COCON) study is to evaluate the (cost-)effectiveness of a nurse-led intervention to optimise implementation of guideline-concordant continence care in comparison with usual care for urinary incontinent women aged 55 years and over who use absorbent products. This paper describes the COCON study protocol.

## Methods

### Design

**Fig. 1:**
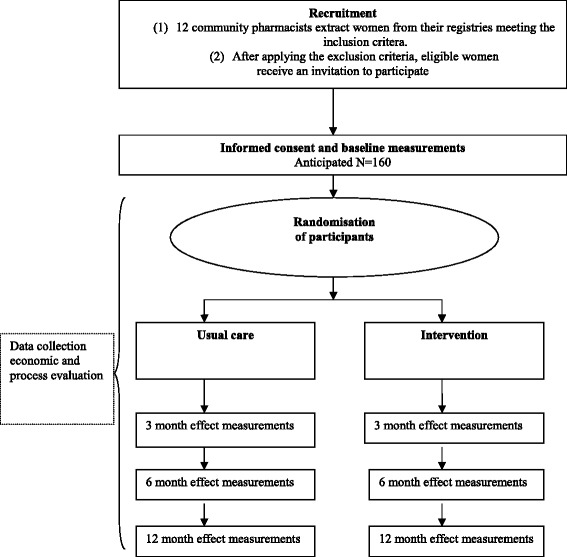
Design of the COCON study

We carry out a randomised clinical trial (RCT) to compare the costs and effects of usual care with guideline-concordant continence care provided by a continence nurse. The trial will run over a 12-month period among women of 55 years and older who suffer from UI and use absorbent products supplied by 12 participating community pharmacies in three Dutch regions (West-Friesland, Katwijk and Amsterdam). We will perform an effect evaluation, an economic evaluation and a process evaluation. Figure 1 shows the design of the study. The COCON study was approved by the medical ethics committee of the VU University Medical Center (approval number 2013.389).

### Study population

The study population consists of community-dwelling and urinary incontinent women aged 55 years and over. All women are long-term users (≥ four months) of absorbent products prescribed by a medical doctor and supplied by a community pharmacy. In addition, all women have sufficient command of the Dutch language to participate in the trial.

#### Participant recruitment

Study participants are recruited by means of the following process.

1. Using the inclusion criteria described above, participating community pharmacies (*n* = 12) provide a list of eligible women. Subsequently, pharmacists exclude women they consider not eligible to participate (due to dementia or cognitive impairment; critical or terminal illness/condition; upcoming transferral to another community pharmacist).
2. By mail, women considered eligible for trial entry receive an invitation to participate in the study. Within two weeks after receiving the invitation, women receive a telephone call from a research assistant who provides further information and asks women whether they are interested to participate.

Finally, participants provide written informed consent and complete the baseline assessment.

#### Randomisation

After obtaining baseline measurements, participants are randomised using an allocation sequence generated by a Random Allocation Software program. The allocation sequence is embedded within the logistic support system built for the COCON study. After baseline measurements, the system issues the group allocation. Stratified block randomisation per community pharmacy is used to ensure an even number of participants in each of the two groups exposed to the same community pharmacist’s policy.

### Control group: usual care

Involvement of continence nurses to stimulate guideline concordant care in women who use absorbent products is not part of usual care in The Netherlands. Usual care for UI is based on guidelines [8, 36]. However, GPs’ adherence to guidelines is generally low [25]. Absorbent product supply and user advice are provided by community pharmacies. Women need to consult their GP before gaining access to continence services (an exception is physiotherapy, which can be accessed without referral) [8]. Monitoring of a woman’s continence status after prescribing absorbent products should be done by the GP [36], but this is rare. The costs of prescription absorbent products are (partly) reimbursed by health insurance companies. The majority of companies base its reimbursement for absorbent products on a fixed day rate that corresponds to clinical user profiles.

### Intervention group: guideline-concordant continence care provided by a continence nurse

During a one year period, participants receive consultations with a continence nurse (on average two hours per year). Based on the Dutch multidisciplinary guideline on UI in women [8], which is in line with international UI guidelines such as the NICE guideline [10], the nurse evaluates and monitors women’s situation and provides advices on treatment and referrals. The nurse advises, whereas the GP decides the (strategy for) UI initial or specialized medical treatment.

To facilitate standardised care delivery, the guideline was translated into a protocol by two experienced continence nurses and a nursing researcher. In the protocol, each health professional’s set of tasks and responsibilities is delineated: responsibility for a patient’s medical treatment rests with the GP, while responsibility for medication safety and absorbent products-related advice rests with the pharmacist. The nurse’s task is to advice a woman about appropriate continence care (including GP diagnostics) tailored to the woman’s specific situation, to support the woman in achieving care agreed on, to evaluate the effects of care, and to monitor continence status. Based on the protocol, an electronic health record system was developed with the aim to compel and monitor compliance with the protocol.

The initial consultation, which takes place at a woman’s community pharmacy or at a women’s location of choice, involves assessment and investigation of (factors related to) continence status to tailor recommendations for treatment (including GP diagnostics) to a woman’s needs and situation (for details, see Additional file 1: Figure S1). Finally, the nurse supports the woman in decision-making on an action plan. Subsequent consultations focus on monitoring continence status and the effects of the action plan agreed on, treatment support, co-ordination of UI care, and evaluation and readjustment of the care agreed on.

There is a minimum of three (at 3, 6, and 12 months of follow-up) and a maximum of six contacts by means of telephone or face-to-face appointments, depending on the woman’s status (see Additional file 1: Figure S1). All consults entail information, education and advice about UI and treatment options. Initially, lifestyle changes and conservative treatment are indicated (e.g., bladder training, pelvic floor muscle training). If these do not work, more invasive treatments can be offered (e.g., surgery). In case of mixed UI, care is directed towards the predominant symptoms (stress or urgency). Bladder diaries are used as an aid in the diagnosis and for the evaluation of treatment [8]. After each consultation, the nurse reports her findings and recommendations, with the woman’s consent, to the woman’s GP. The nurse advises on treatment and care, whereas the GP decides the (strategy for) UI initial or specialized medical treatment. Moreover, the nurse informs the pharmacist about the advice given on absorbent products use.

### Evaluation

Measurements are based on the rationale: guideline-concordant continence care provided by a continence nurse will lead to increased uptake of guideline-concordant care by women. This improved uptake will lead to short-term health benefits in terms of severity of UI, quality of life, and subsequently depressive symptoms. Based on previous studies [28–31], we expect that the maximum benefit will be reached within the first 6 months. In the long term it is expected that the intervention will lead to cost savings due to a decrease in the long-term use of absorbent products. Table 1 provides an overview of all measurements for the effect and economic evaluation.

**Table 1:** Outcomes and measurements of the effect and economic evaluation

Type of outcome	Study entry	Baseline	3 months	6 months	12 months
Patient characteristics	Checking inclusion & exclusion criteria	x			
Severity of urinary incontinence (ICIQ-UI SF)		x	x	x	x
Health-related quality of life (EQ-5D-5 L)		x	x	x	x
Urogenital distress (UDI-6)		x	x	x	x
Improvement of UI (PGI-I)			x	x	x
Depressive symptoms (CES-D)		x	x	x	x
use of absorbent products (in accordance with the recommended criteria in guidelines) (yes/no)		x	x	x	x
*Costs* *(retrospective)*					
questions based on TICP *(past period)*			x	x	x
pharmacist data *(past 12 months)*					x

We perform a process evaluation alongside the trial, collecting qualitative and quantitative data that will assist in explaining and interpreting the findings on (cost-)effectiveness of the intervention. The process evaluation, guided by principles of the Tailored Implementation for Chronic Diseases (TICD) framework [37], will provide insight into the implementation of the intervention. Executed work (Table 2) and user experiences (Table 3) are the main outcomes of interest. Nurses are interviewed at the middle and at the end of the project. Pharmacists and general practitioners are interviewed mid and post project.. In addition, patient satisfaction is measured by means of a post-consultation evaluation questionnaire and an item in the questionnaire at 3, 6, and 12 months of follow-up.

**Table 2:** Work executed

Work to be executed: Intervention components	Concepts	Research questions
- *preparation:* handed over a bladder diary to fill in during three days; asked to take morning urine to the consult - *first consultation(s):* inventory of questions and needs; urine test; bladder diary check; history taking; advice, education and information on UI and appropriate care options followed by shared decision making on care; referral; - *follow-up* consults at 3 and 6 months and at the end of the project, additional consults aligned with the type of UI and care agreed on (according to the protocol) to monitor and evaluate continence status and treatment effects. - *letter to GP* and physiotherapist with advices	Nurses’ delivery of activities, type of activities sequence of activities; collaboration with community pharmacists, GPs and other primary continence care professionals	• What proportion of people received the subsequent intervention components? • How often were the intervention components delivered? • How were the intervention components delivered?
	Nurses’ protocol adherence and deviations; decision making on appropriate treatment advice	• What protocol deviations were necessary? • How was decision making reached? • What changes were proposed and accepted in care for participating women?

**Table 3:** User experiences

Factors	Concepts	Health professionals’ experiences, barriers and facilitators & possible improvements with regard to:
Guideline related factors	Feasibility of the recommended guideline and the protocol derived from the guideline	- Guideline - Protocol - EHR - Consults - Collaboration with other continence professionals: Informing the woman’s GP after the consult by means of a letter, Advices for referrals, diagnostics and treatments
	Compatibility of the protocol: the extent to which the recommended protocol is practical	likewise
Individual health professional factors	Competences (=skills, attitude and knowledge) needed: the extent to which the targeted health professionals have competences they need	- Competences needed - Training/educational needs - Learning experiences
	Health professionals’ engagements and satisfaction with the intervention	- Involvement & Satisfaction (both rated on a 0–10 scale)
Patient factors	Patient behaviour: patient’s response to consults	- Patient’s response to consults, advices - Factors that influence patient’s response to nurses’ care, e.g., financial issues
	Patient motivation: the targeted healthcare professional’s (perceived) ability to motivate patients to adhere	- Motivating patients to adhere
	Patient preferences: the targeted healthcare professional’s (perceived) ability to pay attention to patient preferences	- Paying attention to patient preferences
Professional interactions	Referral processes: processes for communication between the targeted healthcare professionals and targeted patients	- Nurse’s interaction with GPs - Nurse’s interaction with community pharmacists - Nurse’s interaction between GPs and community pharmacists
Incentives and resources	Financial incentives: the extent to which patients, individual health professionals and organisations have financial incentives or disincentives to adhere	- Financial issues that influenced adherence among patients, GPs and community pharmacists
	Information system: the extent to which the EHR facilitates or hinders adherence	- EHR
Capacity for organisational change	Mandate, authority and accountability for making necessary changes	- Mandate for treatment change in participating women.
	Regulations, rules, policies: the extent to which organisational regulations, rules or policies facilitate or hinder necessary changes.	- Regulations, rules and policies.
Social, political and legal factors	Payer or funder policies: the extent to which payer or funder policies may affect implementation of necessary changes	- Payer or funder policies facilitating or hindering implementation of necessary changes
	Legislation: the extent to which legislation may affect implementation of necessary changes	- Legislation affecting implementation of necessary changes

*EHR* Electronic Health Record, *GP* General Practitioner

### Effect evaluation

Primary patient outcome is severity of urinary incontinence as measured with the International Consultation on Incontinence Questionnaire Short Form (ICIQ-UI SF) [38]. The questionnaire has shown good validity, reliability, and responsiveness to change [38].

Secondary patient outcomes are:

1. Health-related quality of life as measured with the five-level version of the EuroQoL (EQ-5D-5L) [39, 40];
2. Urogenital distress as measured with the 6-item Urogenital Distress Inventory (UDI-6) [41];
3. Improvement of UI (in the past period) as measured with the Patient Global Impression of improvement questionnaire (PGI-I) [42];
4. Depressive symptoms as measured with the Center for Epidemiologic Studies Depression Scale (CES-D) [43].
5. Use of absorbent products (yes/no) and use of absorbent products in accordance with the recommended criteria in guidelines (yes/no) which is estimated by questions on whether absorbent products are used as a coping strategy pending definitive treatment, as an adjunct to ongoing therapy, or for long-term management after all treatment options have been explored.

#### Baseline measures

The following patient characteristics are assessed at baseline: age, length and weight (Body Mass Index), parity (number) and type of delivery, UI type, mobility, flatulence and faecal incontinence (yes/no), comorbidity, and treatment history.

#### Sample size calculations

Calculations are based on the expected effects of the intervention on the severity of UI (ICIQ-UI SF), using an equation for a longitudinal design [44]. Assumptions in the calculation were: standard deviation 4.0 [29, 45]; correlation coefficient of repeated measurements 0.6, alpha 0.05; power 95%. To detect a clinically relevant difference of 2 points [45] on the ICIQ-UI SF between the two groups, the number of women needed to include is 64 per arm. Anticipating an attrition rate of 20%, 160 women had to be included.

#### Analysis

To compare the (course of) outcomes between the two groups, mixed model analysis will be performed based on the intention-to-treat principle. First, the characteristics at baseline will be described and the comparability of the groups at baseline will be checked using chi-square tests and student’s t-tests or non-parametric tests. Moreover, potential bias due to differential loss to follow-up will be checked by comparing the baseline characteristics of those with and without loss to the follow-up.

#### Blinding

Due to the nature of the intervention it is not possible to blind participants and nurses. However, data-analysts will be blinded during the analytical process. Pharmacists are blinded to their clients’ allocation status until it is revealed by the nurse or participants themselves. The research assistant and the interviewers cannot remain blinded (for instance, questionnaires in the intervention group contain questions about satisfaction with the intervention).

### Economic evaluation

The economic evaluation will be performed from a societal perspective. Societal costs will be measured retrospectively using questions based on the TICP [46] (see Table 1). Healthcare costs include the costs of GP care, visits to other primary care providers, ambulatory and inpatient hospital care, medication, absorbent products (prescribed products and over-the-counter products), complementary care, and home care. Patient costs will include informal care costs. Lost productivity costs include absenteeism from paid and unpaid work, and presenteeism. Use of absorbent products will be retrieved from the pharmacy’s registration system at the end of the project.

The friction cost approach will be used to estimate lost productivity costs [47]. For the valuation of health care utilisation, standard prices published in the Dutch costing guidelines will be used [48]. Medication costs will be valued using prices of the Royal Dutch Society for Pharmacy [49]. Costs of the intervention will be estimated using a bottom-up approach.

The analysis will be performed according to the intention-to-treat principle. Missing cost and effect data will be imputed using multiple imputation according to the MICE algorithm developed by Van Buuren [50]. Incremental cost-effectiveness ratios (ICERs) will be calculated by dividing the difference in the mean total costs between the two groups by the difference in mean effects between the two groups in (1) severity of UI as measured by the ICIQ-UI SF and (2) quality-adjusted life-years (QALYs) based on the Dutch tariff for the five-level version of the EuroQol questionnaire [39, 51]. Bivariate regression models will be used to estimate cost and effect differences while adjusting for confounding if necessary. Statistical uncertainty will be estimated using bias-corrected accelerated bootstrapping with 5000 replications. Uncertainty surrounding the ICERs will be presented in cost-effectiveness planes and acceptability curves [52, 53]. The policy cost-effectiveness of the intervention will also be estimated using the model developed by Mason et al. [54].

#### Reach of the study

We will explore the reach of the study to gain insight into participants’ representativeness of the target population and associated generalizibility of the results. After recruitment, we collect data among non-participants on the underlying reasons for not participating in the study, and UI-related characteristics (including the primary outcome) by means of a questionnaire. These UI-related data of refusers will be compared with data of participants.

## Discussion

The aim of the COCON study is to evaluate the (cost-)effectiveness of a nurse-led intervention to optimise implementation of guideline-concordant continence care in comparison with usual care for urinary incontinent women aged 55 years and over who use absorbent products.

Results will add to current knowledge of the cost-effectiveness of nurse-led primary healthcare to improve guideline-concordant care for women with UI. In addition, the results will provide more insight into care needs and health service utilization of this group of women, as well as into use of absorbent material in accordance with the recommended criteria in guidelines. Finally, results will increase our understanding of the strategy’s uptake and could provide useful insights for future dissemination and sustenance.

The COCON study differs from previous studies that aimed to improve guideline implementation among women with urinary incontinence in several aspects. First, this study targets only older women with urinary incontinence who are long term users of absorbent products, whereas previous studies focused on all (older) women with urinary incontinence [28, 31, 34]. A second difference is that this study does not rely on GPs [28, 29, 32–35] but is initiated from community pharmacies where women pick up their absorbent products. Third, this study uses a web-based protocol aimed at optimising uniformity of care delivery and facilitating the monitoring of this delivery. Last, this is one of the first studies to investigate cost-effectiveness of guideline-concordant continence care provided by a continence nurse [28, 30].

Strengths of this study include alignment of measurements with the rationale; the absence of potential contamination of the usual care group as this group has no access to the intervention; a process evaluation alongside the trial that will support the interpretation of findings on (cost)effectiveness of the intervention, inclusion of data from women’s consultations with the continence nurse; and insight into (potentially selective) drop-out and refusal of women to enable understanding of the representativeness of participating women and the associated generalisibility of study results.

There are also some limitations of the study. First, selective participation of women is plausible as not all women will accept the consultations with a continence nurse. However, in this study data are collected to gain insight in (potentially selective) drop-out and refusal of women. This will also help to determine which women can be successfully reached by means of this type of strategy in the future. Second, the use of retrospective self-reports on health service utilization might be less valid compared with administrative data. However, as administrative data is not available (with the exception of absorbent products use), self-reports are a good alternative [55].

Despite these limitations, study results will increase our understanding of the uptake and (cost-) effectiveness of nurse supplementation in community continence care. Moreover, we expect that better implementation of the UI guidelines in older women using absorbent products during four months or more can result in both health benefits for women and long-term cost savings for society.

## Trial status

The study commenced recruitment in August 2014 and has completed enrolment of participants in March 2015. The study is currently completing follow-up procedures concerning pharmacist record abstraction. Data cleaning and analysis have not begun. We anticipate that results will be available in March 2017. Some elements of the current study protocol depart from the registered protocol in the Dutch Trial Register (NTR4396): First, we planned a cluster RCT (to overcome potential contamination) and changed this into a randomisation at a patient level as we noticed that there was no risk of contamination at all, and less participants were necessary (160 instead of 252) to execute our study with the same power. Second, initially, we planned to have no exclusion criteria and changed this at request of participating community pharmacists in order to align our study with daily practice. Last, we planned a multifaceted intervention entailing the current intervention and financial incentives for pharmacies contributing to guideline concordant care. We dropped the financial incentives as only the nurse and GP can have impact on uptake of guideline concordant care, not the pharmacists.

## Additional file

Additional file 1: Figure S1. Flow chart nurse’s intervention. (PDF 92 kb)

## Abbreviations

EHR: Electronic health record
GP: General practitioner
UI: Urinary incontinence
