# Supplementary material for: Cost-effectiveness of a nurse-led intervention to optimise implementation of guideline-concordant continence care: Study protocol of the COCON study
Source: BMC Nurs. 2017 Feb 22;16:10. doi: 10.1186/s12912-017-0204-8 (PMC5320796; doi:10.1186/s12912-017-0204-8)
Supplement: Additional file 1: Figure S1. — Flow chart nurse’s intervention. (PDF 92 kb) [file 12912_2017_204_MOESM1_ESM.pdf]

Preparation preceding the first consultation with a woman

- Pharmacist provides nurse with information on woman's absorbent products use in the past half year
- Woman fills in a bladder diary during three days that represent usual variation in her daily activities
- Woman takes her morning urine with her to the consult

First consultation(s), guided by EHR

Nurse's initial assessment and investigation

- Explain role and tasks
- Explore and address the woman's UI-related questions and needs
- Urine dipstick test to detect blood, glucose, protein, leucocytes and nitrites (urinary tract infection)
- Check the bladder diary
- History taking[1]

Depending on the input in the EHR, messages pop-up to remind the nurse to ask additional questions to further clarify the woman's situation and treatment needed.

Nurse's evaluation: clinical reasoning about the woman's situation

Summarize woman's situation:

- Categorise urine incontinence (urinary tract infection suspicion, stress UI, urgency UI, mixed UI, functional UI, overflow bladder)
- Factors that explain and contribute to the complaints
- Factors that should be taken into account in treatment (e.g. comorbidities and limitations)

What else is needed to clarify the situation and care needed?

- GP: requests for additional diagnostics and referrals?

Reasoning on appropriate care: match the patient profile to appropriate care[2]

SUI | UUI | MUI | FUI | OB | UTI

Information, education and advice (tailored to woman's situation/type of UI)

-Etiology of UI (anatomy, pelvic floor muscles)**,** influence of lifestyle on UI, e.g. fluid intake, coffee and alcoholic beverage, weight, constipation, and role of mobility, comorbidity, ageing, and medications.

-Recommendations on lifestyle and healthy toilet behaviour

-BMI > 30: Lose weight (GP request for dietician referral)

Shared decision making (tailored to woman's situation/type of UI)

-Advise on appropriate treatment/care options, as well as GP diagnostics.

-Counsel about prognosis, adverse effects, and (long-term) implications of appropriate treatment/care options.

-Support in deciding on an action plan, register wished care

-If treatment is not possible or rejected, or if absorbent use is indicated, facilitate that woman receive pads that are adapted to her personal needs and leakage.

-letter to woman's GP with the findings, action plan, and advice for GP diagnostics and referrals

-inform pharmacist about decisions on absorbent products use

Monitoring (Follow-up consultations)

- EHR reminds nurse of consultations and provides the summary of the woman's situation and the action plan agreed on
- Monitor: assess UI status, impact of UI, satisfaction with care, GP/professional's response to advices on referrals and diagnostics, evaluate and readjust action plan, build woman's self-confidence
- Information, education and advice (tailored to woman's situation)

Frequency and moments of consultations depend on care agreed on (in any case follow-up consultations take place at 3 and 6 months after shared decision on an action plan ánd at the end of the project)

-letter to woman's GP[3] with the findings, action plan, and advice for GP diagnostics and referrals

-inform pharmacist about decisions on absorbent products use

UI=urinary incontinence; EHR=Electronic Health Record; GP=General Practitioner; SUI=stress urinary incontinence; UUI=urgency urinary incontinence; MUI=mixed urinary incontinence (both stress and urgency urinary incontinence); FUI=functional urinary incontinence; OB=overflow bladder; UTI=urinary tract infection suspicion

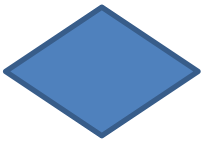

=nurse’s decision on most appropriate care path

[1]**History taking** entails the following subjects:

- UI status and type (urinary tract infection suspicion, stress UI, urgency UI, mixed UI, functional UI, overflow bladder)
- Sandvik index (Sandvik et al., 2000; see below)
- Fecal continence status, obstipation, flatus
- Difficulty emptying
- Urinary tract infection in the past 12 months
- Nocturia
- Absorbent products use
- Skin condition
- Prolapse
- Comorbidities
- Functional and cognitive limitations
- Medication use related to UI
- BMI
- (type of) deliveries and surgery in the pelvic area
- Impact on daily living/quality of life
- Impact on sexuality
- Treatment history and successes

Sandvik Index:

**Quality of urine loss →**	*Drops*	*More than drops*
**Episodes ↓**		
daily	4	8
> 1 time a week	3	6
> 1 time a month	2	4
< 1 time a month	1	2

Sandvik H, Seim A, Vanvik A, Hunskaar S: A severity index for epidemiological surveys of female urinary incontinence: comparison with 48-hour pad-weighing tests. Neurourol Urodyn. 2000, 19 (2): 137-145.

[2] **Advice on appropriate care, additional specifcations per phase per type of urinary incontinence**
The following care paths are distinguished in the protocol: SUI=stress urinary incontinence; UUI=urgency urinary incontinence; MUI=mixed urinary incontinence (both stress and urgency urinary incontinence); FUI=fucntional urinary incontinence; OB=overflow bladder; UTI=urinary tract infection suspicion.
Below, specifications per phase of nurse supplementation are described. In case of mixed urinary incontinence, treatment is directed towards the predominants symtpoms (stress or urgency).

SUI

Information, education and advice (tailored to woman's situation/type of UI)
-GP patient leaflet on stress urinary incontinence

(General) advice on appropriate treatment/care options
General consecutive treatment steps: initial treatment entails lifestyle changes and Pelvic Floor Muscle Training (PFMT),if these are not successful surgery can be considered
Protocolized options:

1) no Pelvic Floor Muscle Training (PFMT) in the past, no contra-indications for PFMT and Sandvik index score: mild or moderate (score $\leq$ 4) → PFMT
2) no Pelvic Floor Muscle Training (PFMT) in the past, no contra-indications for PFMT and a Sandvik index score: severe (score $\geq$ 6) → GP request for surgery
3) PFMT in the past without persistent improvement of complaints → GP request for surgery
4) PFMT contra-indicated due to comorbidities/limitations → GP request for surgery
5) Recent surgery without persistent improvement of complaints or surgery contra-indicated due to comorbidities/limitations → absorbent products and skin products adapted to woman's personal needs and leakage

Monitoring of a care plan agreed on

Agreed on PFMT →
-after 6 weeks a consult by telephone to evaluate effects and to stimulate adherence
-after 3 months: face-to-face consult to evaluate effect of PFMT
in case of insufficient improvement after 3 months: GP request for surgery

Agreed on surgery →
after 6 weeks a consult by telephone to evaluate situation (GP's response, planned surgery) as well as 6 weeks after surgery

UUI

Information, education and advice (tailored to woman's situation/type of UI)
-GP patient leaflet on urge urinary incontinence
-In case of obstipation (which might influence UUI): lifestyle advice, consider GP request for additional diagnostics and drugs to regulate obstipation. After 2 weeks: evaluation of the effect on defecation pattern.

(General) advice on appropriate treatment/care options
*General consecutive treatment steps: initial treatment entails **lifestyle changes** (e.g. cutting down intake of caffeine) and **Bladder Training** (BT). If bladder training does not work, or if it works only partly and the woman still has to pass urine too often, **an anticholinergic drug** can be added to treatment. If this drug is not successful after a month a higher dose or other anticholinergic drug might be considered. If unwanted side effects occur, another drug can be considered. If anticholinergic drugs do not work, treatment with botox, percutaneous sacral nerve stimulation or **surgery** to treat detrusor overactivity can be considered.*
Protocolized options:

1) No bladder training (BT) in the past, no contra-indications (physically or cognitively) for BT → BT
2) BT without persistent improvement of complaints, no contra-indications for anticholinergics use → GP request for additional overactive bladder drugs (anticholinergics) + BT continuation
3) Overactive bladder drugs use without persistent improvement of complaints → GP request for a higher dose of anticholinergics or other type of anticholinergic drug
4) Overactive bladder drugs use (higher dose/other drugs) without persistent improvement of complaints or adverse effects → GP request for Multidisciplinary medical specialist team/surgery, PSNS or botox
5) BT without persistent improvement of complaints and Anticholinergics/overactive bladder drugs contra-indicated → GP request for Multidisciplinary medical specialist team/surgery, PSNS or botox
6) recent surgery without persistent improvement of complaints or surgery contra-indicated due to comorbidities/limitations → absorbent products and skin products adapted to woman's personal needs and leakage

Agreed on BT →
-after 2 weeks a consult by telephone to evaluate effects and to stimulate adherence
-after 6 weeks: face-to-face consult to evaluate effect of BT (Bladder diaries are used as an aid in the the evaluation of the therapy).
-in case of insufficient improvement after 3 months: GP request for overactive bladder drugs (if indicated) or surgery, PSNS or botox.

Agreed on overactive bladder drugs or BT and additional overactive bladder drugs →
-after 6 weeks: a consult to evaluate effects and adverse effects of drugs

Agreed on surgery, botox or PSNS →
-after 6 weeks a consult by telephone to evaluate situation (GP's response, planned surgery) as well as 6 weeks after surgery

MUI

Information, education and advice (tailored to woman's situation/type of UI)
-GP patient leaflet on both stress and urge urinary incontinence

(General) advice on appropriate treatment/care options
Treatment is directed towards the predominant symptoms (stress or urge). In case of comorbidity consider advice on GP referral to a multidisciplinary medical specialist team.

FUI

(General) advice on appropriate treatment/care options
-Advice on how to facilitate removing clothing quickly enough, transferring quickly enough and addressing mobility problems;
-Advice on GP referral to an occupational therapist

OB

(General) advice on appropriate treatment/care options
-GP request for additional diagnostics and treatment; in case of unknown origin: advice on referral to multidisciplinary medical specialist team.

UTI

(General) advice on appropriate treatment/care options & monitoring
-referral to GP (additional diagnostics and treatment for urinary tract infection)
-In case of repeated urinary tract infections (at least 3 infections in a year): GP request for referral to an urologist within a multidisciplinary expert team
-In case of a combination with urge symptoms: 2 weeks after initiation of antibiotics, a consult to evaluate continence status. If urge symptoms till occur and UTI not→ care path of UUI. In letter to GP: request for additional diagnostics preceding the start of anticholinergic drugs, if these drugs are intended.

[3]***Additional issues to be described in the letter to the GP, as well as monitoring of these issues:***

Uncertainty on the type of UI:

- GP request for a referral to a multidisciplinary medical specialist team.

Suspiscion of prolapse and/or difficulty in emptying urine:

- GP request for diagnostics;
- after 2 weeks a consult to evaluate whether prolapse has been validated by the GP or not and to subsequently readjust the care plan;
- in case of a GP referral to a multidisciplinary medical specialist team: consult after 3 months.

Suspiscion of hypertonia of the pelvic floor:

- Referral to a pelvic floor physiotherapist;
- After 2 weeks a consult by telephone to check whether the woman has accepted the advice;
- If pelvic floor physiotherapy has started, after 6 weeks a consult by telephone to evaluate effects and to stimulate adherence; after 3 months a consult to evaluate the effects of the therapy and to readjust the care plan; in case of the absence of persistent improvement of complaints: GP request for a referral to a multidisciplinary medical specialist team.

Suspicion of drug influence on continence status (e.g. antipsychotics, antidepressants, diuretics):

- GP request to consider alternative drugs and, if necessary, to consider a referral to the prescriber of the drug.
